# The Gender Analysis Tools Applied in Natural Disasters Management: A Systematic Literature Review

**DOI:** 10.1371/currents.dis.5e98b6ce04a3f5f314a8462f60970aef

**Published:** 2014-03-18

**Authors:** Sanaz Sohrabizadeh, Sogand Tourani, Hamid Reza Khankeh

**Affiliations:** Department of Health Services Management, School of Health Management and Information Sciences, Iran University of Medical Sciences, Tehran, Iran; Department of Health Services Management, School of Health Management and Information Sciences, Iran University of Medical Sciences, Tehran, Iran; 3. Hospital Management Research Center (HMRC), Iran University of Medical Sciences, Tehran, Iran; Department of Health in Emergency and Disaster, University of Social Welfare and Rehabilitation Sciences, Tehran, Iran; Department of Clinical Science and education, Karolinska Institute, Stockholm, Sweden

## Abstract

Background: Although natural disasters have caused considerable damages around the world, and gender analysis can improve community disaster preparedness or mitigation, there is little research about the gendered analytical tools and methods in communities exposed to natural disasters and hazards. These tools evaluate gender vulnerability and capacity in pre-disaster and post-disaster phases of the disaster management cycle. Objectives: Identifying the analytical gender tools and the strengths and limitations of them as well as determining gender analysis studies which had emphasized on the importance of using gender analysis in disasters. Methods: The literature search was conducted in June 2013 using PubMed, Web of Sciences, ProQuest Research Library, World Health Organization Library, Gender and Disaster Network (GDN) archive. All articles, guidelines, fact sheets and other materials that provided an analytical framework for a gender analysis approach in disasters were included and the non-English documents as well as gender studies of non-disasters area were excluded. Analysis of the included studies was done separately by descriptive and thematic analyses. Results: A total of 207 documents were retrieved, of which only nine references were included. Of these, 45% were in form of checklist, 33% case study report, and the remaining 22% were article. All selected papers were published within the period 1994-2012. Conclusions: A focus on women’s vulnerability in the related research and the lack of valid and reliable gender analysis tools were considerable issues identified by the literature review. Although non-English literatures with English abstract were included in the study, the possible exclusion of non-English ones was found as the limitation of this study.

## Introduction

During the past four decades, natural disasters such as earthquakes, droughts, floods and storms have caused a considerable loss of human lives and livelihoods, environmental damage and the destruction of economic and social infrastructure^1^. Since disaster can be the result of a combination of natural hazards and people’s vulnerabilities, there is an urgent need to reduce disaster impacts, which can only be done by shifting the typical paradigm from an exclusive emphasis on disaster response to comprehensive disaster risk reduction 2. People’s vulnerability is determined by physical, social, economic and environmental factors, but socio-economic factors are more closely related to vulnerability than other factors^3^^,^ 4.

Gender is an important variable among social factors. It is a major dimension of social difference, and cultural norms can help people understand various degrees of vulnerability 4 ^,^ 5 ^,^ 6. Thus, understanding vulnerability and developing strategies to overcome it can be promoted through a gender analysis tool that can contribute significantly to addressing the root causes of vulnerability 7.

Although gender plays a major role in the participation, involvement, resource allocation, disaster mitigation and preparedness decision making 5, it has been ignored as an analytical tool and disaster research approach. In much disaster research, gender is simply a quantitatively measured background characteristic rather than a central analytical element 4, and only a few studies contained a comprehensive analysis of the gendered vulnerabilities and capacities of an affected population 8. In addition, there is often a lack of gender-sensitive indicators that can be used to evaluate the outcomes of gender-focused policies, assess challenges to success and adjust activities to reduce the adverse impacts of disasters on women and men 9.

There are existing gaps in gender analysis, and little is known about analytical tools or indicators to address the different needs of women and men and evaluate gender vulnerability and capacity in pre-disaster and post-disaster phases of the disaster management cycle. Therefore, this study aims to determine the analytical gender tools and to assess the strengths and limitations of them. In addition, this study aims to determine the reports of gender analysis projects in natural disasters fields as well as to identify the studies which had emphasized on the importance of using gender analysis in disasters. The included documents will identify gaps that can be filled by further research and hasten the development of different interventions and strategies to address the types of action to be taken. Thus,

**This paper adds:**

1- A collection of international research on gender analytical tools and indicators to assess gender vulnerability and capacity before, during and after disasters

2- Recommendations for future gender research, which will lead to implementing effective interventions in disaster areas.

## Method

***Databases and Search Strategy*** **

This study is a systematic review; the literature search was conducted in June 2013 using the following databases:

- PubMed

- Web of Science

- ProQuest Research Library

- World Health Organization Library

- Gender and Disaster Network (GDN) archive

The PubMed and WHO libraries were searched for gender analysis indicators or tools related to health outcomes of disasters. On the other hand, Web of Science and ProQuest were used to attain gender indicators or tools related to other effects of disasters such as social, economic and environmental aspects. The GDN archive was used as a supplemental database and has been identified as one of the most comprehensive resources related specifically to gender approach in disasters.

The search terms and keywords were selected after consulting with the disaster sociology and disaster management researchers. The main search terms were applied in four parts: 1- keywords related to disasters, 2- the terms related to gender, 3- the keywords related to disaster risk management and 4- the terms related to evaluation or analysis.

The controlled vocabulary of Medical Subject Headings (MeSH) from PubMed was used, when applicable, to adjust and control the terms and search the databases. It ensured a controlled vocabulary, even in databases that do not use MeSH to index articles. In addition, the search strategy of the PubMed database was used as a model to search the other databases (table 1). The search strategies of all databases were checked and revised by the health information specialist and according to his revision the final search strategies were modified. The table illustrates the applied keywords used to search the databases.

**Table 1 Selected databases with the search strategies:** 

**Database**	Search strategy
*PubMed*	(disaster*[tiab] OR emergencies[tiab] OR Earthquake*[tiab] OR Flood*[tiab]) AND (“gender identity”[Mesh] OR “Femininity” [Mesh] OR “Masculinity” [Mesh] OR gender*[tiab] OR sex[tiab] OR women[tiab] OR men[tiab] OR Femininity* [tiab] OR Masculinity* [tiab] OR female*[tiab] OR male*[tiab]) AND (vulnerab*[tiab] OR capacit*[tiab] OR empower*[tiab]) AND (indicat*[tiab] OR factor*[tiab] OR ind*[tiab] OR measure*[tiab] OR assess*[tiab] OR manag*[tiab] OR evaluat*[tiab] OR analy*[tiab])
*Web of Science*	TS=(natural disaster* OR natural hazard OR Earthquake* OR flood* ) AND TS=(gender* OR sex OR women OR men OR Femininity* OR Masculinity* OR female* OR male*) AND TS=(vulnerab* OR capacit* OR empower*) AND TS=(indicat* OR factor* OR index OR measure* OR assess* OR manag* OR evaluat* OR analy*)
*ProQuest*	ab((natural disaster* OR natural hazard OR Earthquake* OR flood*)) AND ab((gender* OR sex OR women OR men OR Femininity* OR Masculinity* OR female* OR male*)) AND ab((vulnerab* OR capacit* OR empower*)) AND ab((indicat* OR factor* OR index OR measure* OR assess* OR manag* OR evaluat* OR analy*))
*WHO*	(natural disaster OR earthquake OR flood) AND (gender OR women OR men) AND (vulnerability OR capacity) AND (indicator OR index OR factor) AND (assessment OR evaluation OR measurement)

Depending on the database type, there were different limitations used in the searches. For example, the document type was selected as article in PubMed, Web of Science and ProQuest Research Library, but this limitation was not implemented when searching the WHO library and the GDN archive. The publication years included articles between 1990 and June 2013 in PubMed and Web of Science, and 1995 to June 2013 in ProQuest because the concept of gender mainstreaming in disaster management has been developing for about 20 years.^3^ The other two databases did not have a publication year limitation.

***Inclusion and exclusion criteria*** **

For the selection of relevant studies, the research team applied two different sets of criteria identified as inclusion and exclusion criteria, explained below:

- Inclusion criteria:

- Worldwide articles, guidelines, fact sheets and other materials that provided an analytical framework for a gender analysis approach in disasters.

- International articles, guidelines, fact sheets and other materials that introduced the tools or indicators of gendered vulnerability and capacity analysis in disasters.

- Exclusion criteria:

- All non-English literature

- Literature without an available abstract

- Gender studies of non-disaster areas

***Studies selection*** **

The titles of all identified papers were scanned by the principal author, and only the studies that clearly met one or both of the inclusion criteria were selected for the next stage of screening. Additionally, the studies that met one of the exclusion criteria were rejected. The abstracts and full texts of all selected literature were then investigated separately by two review authors, and any disagreements related to the studies selection were resolved through consensus or by consultation with a third member of the review team. Data extraction of all selected articles was performed using a pre-defined checklist created by the authors.

***Data analysis*** **

Once the search process for studies selection was completed and the relevant papers had been selected, descriptive and thematic analyses were done separately. A descriptive analysis clarified the main characteristics of the selected literature such as the type of paper, the kind of disasters and the methodologies used. The thematic analysis was done to synthesis the main outcomes extracted from the relevant studies, such as categorization of gender analysis tools or indicators in disasters. The main purpose of the thematic analysis of analytical gender tools was to inform future research and assessment practice. This review is reported according to PRISMA guideline.

## Results

The initial search generated 207 potentially relevant references. After removing duplicates, 195 references were identified for screening. Upon further review, 51 references that met the exclusion criteria were eliminated. According to an abstracts analysis of the remaining 145 references, 21 studies were selected and the full text was read. Finally, 9 key studies were included in the review (figure 1). The results were divided into a descriptive and thematic analysis.

**Flow chart of articles selection process:**
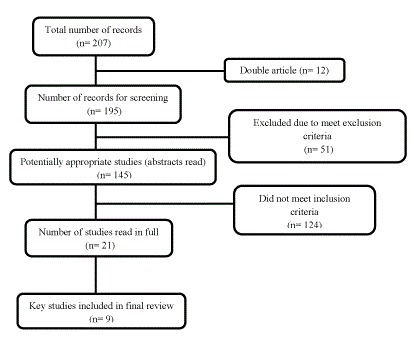
The process of final studies selection illustrated in the flow chart

***Descriptive analysis*** **

The descriptive analysis aims to provide an overview of the characteristics of the included studies. The studies selected for the systematic review were recorded by:

- The year of publication within the period 1994-2012

- The type of studies

- The summary of all selected studies

Table 2 shows the author name, the year of publication, and the summary of all selected studies; figure 2 illustrates the different types of studies. Each issue is briefly discussed: Descriptive analysis of the selected documents showed that all selected papers were published within the period 1994-2012 and the highest number of publications was in the years 2001 and 2004. Additionally, the most-included document type was in the form of a checklist that makes data gathering possible (45%). Many studies came from Latin and Central America.

**Table 2. Included studies for final analysis:** 

**Author**	year	Title	Document characteristics
World Health Organization (WHO)	2005	Gender consideration in disaster assessment	The checklist consisted of two key parts: key questions of gender analysis in disasters and principals of good practice
Rozan	2006	Checklist to facilitate gender sensitivity of relief and reconstruction efforts for survivors of the earthquake in Pakistan	The checklist arranged in three sections including an introduction, key questions and supplementary information on possible challenges and opportunities
Deare	2004	A methodological approach to gender analysis in natural disaster assessment	The document provided tools and methodologies to conduct gender analysis in the Caribbean region in pre-disaster and post- disaster situation
Sequeira	2001	Risk management: an alternative perspective in gender analysis	The paper agued principle opportunities to factor gender considerations into risk management with the mention of risk scenarios and experiences from Central America
Bradshaw	2001	Reconstructing roles and relations: A gendered analysis of women's participation in reconstruction in post-Mitch Nicaragua	The paper reported the results of the Social Audit and in depth study undertaken in the households of four affected communities with emphasis on the roles of women in reconstruction phase
Bradshaw	2004	Socio-economic impacts of natural disasters: A gender analysis	The paper analyzed the socio-economic effects of hurricane Mitch using a gender approach and suggested new analysis indicators for crisis situations in the four sections
Anderson	1994	Understanding the Disaster-Development Continuum: Gender Analysis is the Essential Tool	The article explained the importance of gender analysis as the key element of connecting disasters and long - term development
Enarson	1999	Gender – aware disaster practice: A self assessment tool for disaster responding agencies	The checklist made up of the three sections with key questions to help the identification and assessment of gender issues in the response agencies
Eklund and Tellier	2012	Gender and international crisis response: do we have the data, and does it matter?	The article reviewed available analyses, assessments and academic literature to gain insights into whether sex - disaggregated data are generated

**Classification of selected studies according to the type of documents:**
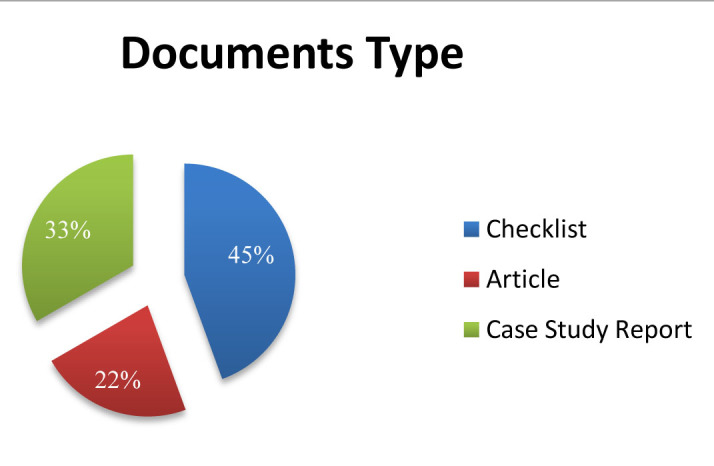
Included documents types illustrated in three parts: checklist, article and report.

***Thematic analysis*** **

The thematic analysis is as follows: review of the gender analysis tools or checklists; reports of gender analysis implementation; and articles that explained the importance of gender analysis tools or checklists in disasters.

***1- Gender analysis checklists:***

In response to the lack of gendered data in disaster assessment, the WHO (2005) prepared an applicable checklist for gathering gender-disaggregated data in disaster fields. The checklist includes two main parts: the key questions and the principles of good practice. The key questions are divided into different categories, such as specific needs of women in a tsunami aftermath; women as caregivers; access to aid; and vulnerability to exploitation/abuse. Women’s situations and their vulnerability and capacity in disasters are the focus of most of the key questions in the checklist. The principles of good practice are revealed at the end of the tool as well, but cover considerations for both women and men in disaster assessments such as decision-making involvement, providing access to supplies and documentation of gender-disaggregated data.^10 ^It seems that this checklist considers some useful and practical health points for assessing the women and men needs in disasters such as tsunami. Although, it can be used for the health assessment as a part of comprehensive gender analysis, there is the lack of existing the important aspects of public health in details (reproductive health, environmental health and etc.) for all natural hazards.

Another gender analysis checklist developed by Rozan (2006) in Pakistan facilitates gender integration in different aspects of relief and recovery, with a focus on six major clusters: education; water and sanitation; protection; shelter and camp management; health and nutrition; and early recovery and reconstruction. Each cluster provides key questions about specific needs, vulnerabilities and capacities of women and men. Additionally, each cluster is categorized by some key concepts; for example, the education cluster includes access to schools, educational and recreational content, a reporting system for harassment and abuse, staff recruitment and training and educational and vocational activities for adults. The water and sanitation cluster was classified into access to resources, special health/sanitation needs, community participation and staff training.^11^ In total, this checklist is one of the comprehensive checklists with all social, economical and health parts of gender analysis in disasters; however, the checklist highlights the women needs and vulnerabilities in disasters more than men. The possible challenges and opportunities for each cluster of gender analysis in the final section of the checklist can be considered as another advantage of the checklist for comprehensive gender analysis in disasters.

One gender analysis checklist was developed by Enarson (1999) to help disaster planning and response agencies assess gender issues. There are three main parts in the checklist: staff and training, program development and community outreach. Each part has some questions that place more emphasis on women’s situations in pre- and post-disaster phases and considers vulnerable and at-risk women as well.^12 ^ It seems that the assessment of women needs and capacities in disasters agencies need paying more attention from the disaster managers and it’s better to apply such checklists for gender analysis in the responsible organizations for disasters management.

Deare (2004) prepared a useful document with tools and methodologies for gender analysis in disaster assessment of the Caribbean region. This document presents an analysis of the socio-economic effects of natural disasters from a gender perspective and a methodological framework for gender analysis. Gender analysis of data gathering methods, evaluation of pre-disaster and post-disaster gender relations, gender analysis of health and social network response and examination of gender aspects in resource allocation are the main concepts discussed in this document. The study explained two methods of gender-disaggregated data collection during the assessment period: secondary data sources and primary data sources. The information obtained by secondary resources such as statistical offices or regional databases may not be gender-disaggregated or available like health status, social and political structure, ethnic and cultural patterns and communications. Thus, primary data collection with a rapid appraisal method should be used to collect adequate information through a combination of semi-structured and focus group interviewing, resource mapping, transect walks and participant observation. The evaluation checklist for pre-disaster gender relations provides some gender baseline information such as housing, education systems and social and health systems. An assessment checklist of post-disaster gender impact includes an analysis of vulnerable groups using a special matrix with two main parts: resources (physical, socio-economic and organizational) and vulnerable groups disaggregated by gender (households with large families, farm workers, seniors living alone and squatters) 13. It seems that this full document is the best one with a lot of aspects of gender analysis in disasters but it has been developed for Caribbean region and we are not sure if it can be used for other different regions such as Asian countries. The most important limitation of all above-mentioned checklists is the lack of reliable and valid tool development. None of authors have developed the standardized checklist by scientific methodology such as panel of experts for content validity, factor analysis for structure validity and test – retest for ensuring about reliability and etc.

***2- Gender analysis reports***

Bradshaw (2001) reported a gendered analysis of women’s participation in reconstruction after Nicaragua was hit by Hurricane Mitch, using the results of a Social Audit and a study conducted in four affected communities where the men and women were interviewed. The Social Audit also collected information about both the psychological effects of Hurricane Mith on women and violence against women in the community. The investigation of gender impact after Hurricane Mitch showed that three quarters of the emotionally affected people were women or girls. During the crisis, women were performing their traditional roles and also worked with men to evacuate people, move belongings, clear the roads and create safe passages. Men viewed the women’s traditional activities as ‘doing nothing’. In addition, a decline in the number of women in productive work was seen in long-term reconstruction after Hurricane Mitch affected their access to and control over resources. The lowest levels of participation were recorded among young female partners/wives with male heads of households, and the highest levels were among female heads of household .^14^

Sequeira (2001) explained the importance of gender analysis perspective in risk management and discussed the issues related to gender consideration in disaster risk management in Central America. First, the study explained the natural disasters in Central America and the importance of vulnerability reduction and capacity building to decrease the risk of disasters. Second, the problems in risk reduction plans based on gender consideration were revealed, including design and implementation of training programs without a gender approach; the lack of gender analysis in the community; a focus on understanding the complexity of gender relations instead of a simple and clear strategy such as giving a consultation to a few women .^15^

Bradshaw (2004) analysed the socio-economic effects of Hurricane Mitch with gender consideration to reflect women’s disadvantageous position relative to men. The first part of the study explained the key concepts of gender analysis and disasters in the context of Hurricane Mitch and also demonstrated women’s vulnerability prior to Mitch. The next sections investigated the direct and indirect impacts of disasters on women. Material damage disaggregated by gender (loss of life, housing, social infrastructure, productive labour and community work) and recognition/lack of recognition of work carried out by women, were considered direct impacts. Indirect impacts included migration, which leads to a rise in the number of female-headed households, psychosocial impact (feelings of fear and insecurity) and violence against women. The response to Mitch with gender considerations was conducted at three levels: coping strategies to deal with the crisis; the actions of governments and the coordinated bodies of civil society; and the reconstruction initiatives implemented by national and international organizations (national plan) .^16^

The above mentioned documents showed the gender analysis research projects in the field of disasters. However, the tools of gender analysis have not been completely described by these researchers. The studies reveal some important issues and lesson learned to help conduct future gender analysis research around the world. They can be useful for developing the future comprehensive gender analysis checklists for disasters management as well.

***3- The importance of gender analysis tools in disasters***

Anderson (1994) linked the disaster development continuum to gender analysis as an essential tool that can contribute significantly to addressing root causes and can support effective and equitable long-term development. The article discussed that an understanding of vulnerability and the strategy development to overcome it can be achieved through gender analysis. The article said that women are more vulnerable than men because of their traditional roles, and understanding the causes of their vulnerability can help to identify ways in which men are also vulnerable. Eklund and Tellier challenged the lack of gender-disaggregated data in the crisis response phase. The article’s findings revealed that humanitarian response evaluations rarely collected or referred to gender-disaggregated data, and there is a gap between policy and practice in this field. According to the article, important information to reduce the vulnerability of both males and females can be obtained from existing data and population-level studies related to the disaster situations .^7^^,^ 8

## Discussion

This systematic review of gender analytical tools highlights a number of problems, and the gaps in knowledge about gender analysis in disasters and the number of gendered analytical tools or checklists to conduct gender analysis surveys before and after disaster occurrences should be researched further. In particular, the findings of the descriptive and thematic analyses can be discussed and implications for both the scholars and the practitioners who operate in the field of gender approach in disaster management can be suggested.

The descriptive analysis of the selected studies indicates that gendered analytical checklists were rarely identified or used by most of the disaster managers and scholars around the world. Gender analysis, however, is a relatively recent issue in disasters, which can explain the lack of assessment tools related to this issue. On the other hand, it seems that some regions such as Latin America are more active in creating a methodology of gender analysis and conducting gender analysis with suitable checklists in the pre- and post-disaster phases. The number of scholars or practitioners in Latin America who are active in gender analysis performance in disasters is considerable, or maybe, compared with other gender analysts, they highly report or publish their gender analysis projects or checklists on international electronic databases. The descriptive analysis suggests some conclusions:

**• **The number of documents related to gender analytical tools or checklists in disaster assessment and evaluation has not increased significantly in recent years. Thus, it can be proposed that contributions to the literature should focus on sharing the applied methods of gender analysis in the disaster-affected population.

**• **More contributions come from the Americas, while contributions from Asia are still modest. Because the results of gendered vulnerability and capacity analysis are context-based and will change according to the disaster-affected region, the tools or checklists designed in one area may not be useful for others. One suggestion is to make gender analytical tools at the local level a priority for all regions, especially the high-risk areas, to manage future disasters efficiently.

The thematic analysis of the literature review was conducted in three parts, including checklists or methodological tools; reports of gender analysis before and after disasters; and implications related to the importance of gender analysis tools in disaster assessment. The thematic analysis of gender analytical tools included in the study indicates that the development of most checklists or methodological tools is based on women’s vulnerability. The capacities of women in traditional and non-traditional roles are not noticed enough. It seems that there is more emphasis on women’s vulnerability at international and local levels than on women’s capacity, and this viewpoint is reflected in the development of checklists or tools for gender analysis in disaster assessments. The lower physical ability of women compared to men may affect their potential capacities, which leads researchers to classify women into vulnerability groups in much of the literature. Thus, it can be suggested that the development of gender analytical tools and gender analysis implementation in all phases of disaster management should demonstrate the balance of both women’s capacity and vulnerability to handle the unsuitable situations in disasters.

On the other hand, the men’s vulnerability and capacity analyses are rarely highlighted in the gender analysis tools. Although gender consists of both women and men, most of the related documents have focused on women’s needs and work in disaster evaluation, and men’s considerations in some reports are depending on their actions which have affected women.

The selected documents that reported the findings of the gender analysis project related to different phases of disaster management, such as recovery or mitigation, did not usually refer to a useful analytical tool or checklist in their methodology (the exception was gender analysis in the Caribbean region). However, sharing experiences and lessons learned by gender analysis performance in a variety of regions can be useful for other disaster researchers and scientists.

The thematic analysis suggested the following for future research and practice:

**• **Development of valid and reliable gender analysis tools in disasters to assess and evaluate the gendered vulnerability and capacity efficiently and accurately.

**• **Designing and setting gender-based databases at the local, national and international levels that can be used as the centre of gender-disaggregated data collection for gender analysis before and after disasters.

**• **Community-based gender analysis with the participation of both men and women.

## Limitations

The main limitation of this review is the fact that only English language documents were included in the study and other non-English ones were excluded. Although the non-English literatures with English abstract were selected and studied, it is likely that we have missed other potential related studies which can lead to incomplete coverage of studies in other languages. Another limitation was the lack of clear terminology for gender. Although we extended our search strategy by related different keywords using MeSH database, some relevant tools or studies with uncommon keywords may likely be missed.

## Conclusion

The importance of gender analysis and gender-disaggregated data collections for efficient and suitable assessment and evaluation in all stages of disaster management has been explained in many studies. The gap between theory and practice can be seen in the field of gender analysis, but there is a need to simultaneously strengthen both the theory and practice of gender approach in disasters. It is necessary to ensure that all needs and abilities of men and women have been met before, during and after disasters.

## Correspondence

Sogand Tourani

E- mail: tourani@hmrc.ir

## Appendix 1

### PRISMA Checklist

Download PDF
